# Increased serum oxidized low-density lipoprotein levels in pregnancies complicated by gestational diabetes mellitus

**Published:** 2015-07

**Authors:** Azam Ghaneei, Sara Yassini, Mohammad Ebrahim Ghanei, Ahmad Shojaoddiny-Ardekani

**Affiliations:** 1 *Department of Endocrinology, Shahid Sadoughi University of Medical Sciences, Yazd, Iran.*; 2 * Shahid Sadoughi University of Medical Sciences, Yazd, Iran.*; 3 *Radiologist, Isfahan University of Medical Sciences, Isfahan, Iran.*; 4 *Diabetes Research Center, Shahid Sadoughi University of Medical Sciences, Yazd, Iran.*

**Keywords:** *Oxidized low*-*density lipoprotein*, *Gestational diabetes*, *Pregnancy*, *Body mass index*

## Abstract

**Background:**

Elevated serum levels of oxidized Low-density Lipoprotein (oxLDL) have been found in type 2 and in poorly controlled diabetic patients. Gestational diabetes mellitus (GDM) has common features with type 2 diabetes.

**Objective:**

The aim of our study was to evaluate the serum levels of oxLDL in women with GDM compared to normal pregnant women.

**Materials and Methods:**

In this cross-sectional study, ninety-two subjects were randomly allocated to either GDM (n=46) or control (n=46) groups matched for age, body mass index and parity from March 2013 to March 2014. GDM was diagnosed according to the American Diabetes Association criteria at 24-26 weeks of gestation. OxLDL was measured using enzyme-linked immunosorbent assay. T-test and Pearson correlation coefficients were applied for analyzing the data by using SPSS version 17.

**Results:**

Compared to the controls, significantly higher oxLDL levels were found in the GDM group (17.16 ± 3.71 U/L vs. 8.77 ± 1.84 U/L, respectively, p < 0.001). No significant correlations were found between oxLDL and age and BMI of the patients in the groups.

**Conclusion:**

Our study found significant increase of oxLDL in GDM emphasizing the role of short-term hyperglycemia in the formation of oxLDL during GDM. The importance of aptly diagnosis of GDM in maternal health may also be concluded.

## Introduction

Gestational diabetes mellitus (GDM) is defined as carbohydrate intolerance which firstly initiates or is diagnosed during pregnancy (1). It is a worldwide medical complication affecting about 7 percent of pregnancies (2).

Modified maternal lipoprotein profile, increased oxidative stress and oxidized products of lipid peroxidation namely oxLDL have been found in human placenta during pregnancy. This process is considered a principal step for the development of atherosclerosis (3). In addition, elevated levels of oxidized Low-density Lipoprotein (oxLDL) have been found in type 2 diabetic patients (4-7). GDM has common features with type 2 diabetes mellitus; there are similar risk factors, mechanisms (i.e. insulin resistance and impaired insulin secretion) and genetic susceptibility for the both disorders (8). Increased levels of progestogens, hypercholesterolemia and hyperglycemia which occur during GDM accelerate the LDL oxidation (9). 

Although there are studies evaluated the oxLDL in type 2 diabetes, limited studies assessed it in women with GDM. So, the aim of this study was to evaluate the serum levels of oxLDL in women with GDM in comparison with normal pregnant women.

## Materials and methods

In this analytic cross-sectional study, from patients admitted at the outpatient endocrine clinic of Shahid Sadoughi hospital, Yazd, Iran 46 newly diagnosed pregnant women with GDM and 46 healthy pregnant volunteers who were matched for age, parity and pre-pregnancy body mass index (BMI) studied from March 2013 to March 2014. GDM was diagnosed according to the American Diabetes Association criteria (10) at 24-26 weeks of gestation. Based on these criteria, the diagnosis of GDM is made when any of the following plasma glucose values are exceeded: fasting: ≥92 mg/dL, 1 h: ≥180 mg/dL, and 2 h: ≥153 mg/dL. GDM also has been ruled out in them at their first prenatal visit and GDM group merely consisted from the patients who firstly diagnosed with GDM at 24-26 weeks of gestation. The following variables were determined for each patient: age, height, weight, parity, fasting plasma glucose, 1-hr plasma glucose, 2-hr plasma glucose, serum creatinine and oxidized LDL. Weight was measured with a digital scale (measurement accuracy of 100 g), with subjects in minimum clothing and without shoes. Height was measured by Seca Scales (Seca 216 Stadiometer, Scales Galore Co., US) and height measuring systems with subjects standing shoeless and with their shoulders set normally. BMI was then calculated by dividing weight (kg) by the squared height (m^2^). Women were advised to follow a normal diet 48 hours before the oral glucose-tolerance test (OGTT) and to fast for 8-14 hours the night before the test. Blood samples were obtained after the overnight fast and one and two hours after the receipt of the 75-g oral glucose load. OxLDL was measured using commercially available sandwich enzyme-linked immunosorbent assay (Mercodia, Uppsala, Sweden). The intra- and inter assay CV for the assay ranged between 5.4% and 8.3%. The detection limit was 0.03 U/L. Women with hypertension, preeclampsia, hepatic diseases, congestive heart failure, thyroid disorders, proteinuria or renal involvement (creatinine >1.5 mg/dL or glomerular filtration rate <70 mL/min), were excluded. The study protocol was approved by the medical ethics committee of Shahid Sadoughi University of Medical Sciences, Yazd, Iran. Written informed consent was taken from all the participants.

**Statistical analysis**

Statistical analysis was performed by using SPSS software (Statistical Package for the Social Sciences, version 17.0, SPSS Inc, Chicago, Illinois, USA). The continuous variables are expressed as mean ± standard deviation. Comparison between patients and controls were performed by T-test for quantitative variables. Pearson correlation coefficients between oxLDL and other continuous variables were calculated. P-value < 0.05 was considered significant.

## Results

Ninety-two subjects were randomly allocated to either GDM (n=46) or control (n=46) groups. Table I represents demographic and biochemical characteristics of the study groups. As seen in the table, there were no significant differences between the groups with regard to their age, BMI and parity. Compared to the controls, significantly higher oxLDL levels were found in the GDM group (17.16 ± 3.71 U/L vs. 8.77 ± 1.84 U/L, respectively, p < 0.001).

Pearson correlation analysis of the variables in GDM and control groups showed no significant correlations between the oxLDL and the age and BMI of the patients in the GDM group (r=0.273 and -0.154, respectively). The relationship between age and BMI was also not significant (r=-0.019). Similarly, in the control group, the coefficients (r) for the correlation between oxLDL and age and BMI were 0.215 and -0.264, respectively. There was not significant relationship between age and BMI of the controls as well (r=-0.149).

Table II shows the relationship between oxLDL levels and plasma glucose levels as determined by OGTT in the study groups.

**Table I:** Comparison of the characteristics of the study groups

**Variables**	**GDM group (n=46)**	**Control group (n=46)**	**p-value**
**Age (year)**	25.67 ± 4.81	25.52 ± 4.56	0.877
**Body Mass Index (kg/m2)**	28.88 ± 3.07	29.59 ± 2.72	0.247
**Parity (n)**	2.30 ± 1.01	2.35 ± 1.08	0.842
**OxLDL (U/L)**	17.16 ± 3.71	8.77 ± 1.84	<0.001*

Data are presented as Mean±SD.

OxLDL: Oxidized low-density lipoprotein.

GOM: Gestational diabetes mellitus

* Significant

**Table II:** Relationship between serum oxLDL levels and plasma glucose levels during OGTT in the study groups.

**Groups**	**Test**	**r**	**p-value**
**GDM (n=46)**	Fasting Plasma glucose	0.08	0.56
	1-hr plasma glucose	0.24	0.1
	2-hr plasma glucose	0.15	0.29
**Control (n=46)**	Fasting plasma glucose	0.07	0.6
	1-hr plasma glucose	0.04	0.75
	2-hr plasma glucose	0.3	0.04*

OxLDL: oxidized low-density lipoprotein.

OGTT: Oral glucose tolerance test.

GDM: Gestational diabetes mellitus

r: Pearson correlation coefficient.

* Significant

## Discussion

To the best of our knowledge, this is the first study evaluating the serum oxLDL levels in pregnancies complicated by GDM. Our results showed higher serum level of oxLDL in GDM patients compared to healthy pregnant participants. There was no association between oxLDL levels and demographic variables such as age and BMI. These findings are in agreement with previous evidences showing increased oxidative stress in diabetic patients.

Toescu et al, showed that diabetic pregnancy is associated with the changes in serum lipids normally consistent with increased risk of coronary artery disease as well as the evidence of increased oxidative stress compared to non-diabetic pregnancy (11). The placenta is a cornerstone supporting organ in fetus development and acts as the site of exchange for lipids (14). Physiological adaptations occurring during pregnancy alter maternal lipoprotein metabolism which in turn results in increased levels of lipoproteins from second trimester to the time of delivery (13).

A history of GDM increases the risk of further developing of type 2 diabetes mellitus as well as cardiovascular disorders. GDM and type 2 diabetes mellitus share some characteristics including risk factors, pathophysiological mechanisms, and genetic susceptibility (8). Indeed, elevated levels of oxLDL have been found in type 2 diabetic patients (4-7). Dyslipidemia which is frequently seen in GDM favors insulin resistance and LDL oxidation and could damage the placenta and thus leads to adverse pregnancy outcomes such as intrauterine growth restriction (14). Increased progestogens, hypercholesterolemia and hyperglycemia are of factors accelerating the LDL oxidation; these conditions occur during GDM. In vitro studies have demonstrated the effect of high glucose concentrations in the acceleration of LDL oxidation both in isolated LDL preparations and in cell culture model (9).

Studies show that hyperglycemic status leads to non-enzymatic oxidation of lipids and proteins. Increased reactive oxygen species (ROS) level also causes insulin resistance in peripheral tissues while deteriorating insulin secretion from islet cells (15). A proposed mechanism is impaired translocation of intracellular vesicles containing glucose transporter 4 (GLUT4) to the plasma membrane of adipocytes and myocytes following stimulation by insulin in the presence of oxidized LDL (16). Furthermore, in GDM pregnancies, there is increased placental protein expression of lectin-like oxidized LDL receptor-1 (OLR1) compared to normal lipid profile pregnancies; this may be associated with GDM pathology (13).

It is also shown that GDM increases LDL susceptibility to oxidation even before the diagnosis of diabetes. This susceptibility is related to diabetes per se, but also is due to higher incidence of obesity and hypercholesterolemia in these women (14).

The oxidized form of LDL is now considered a key component in the development of atherosclerosis. In the study done by Gokulakrishnan et al. in India, mean oxLDL levels were significantly higher in subjects with diabetes and impaired glucose tolerance compared with normal glucose tolerance group (p <0.001); furthermore, oxLDL was associated with carotid intimal medial thickness (17).

In the study by Nakhjavani et al. Serum oxLDL level was significantly higher in patients with prolonged diabetes in comparison with newly diagnosed diabetic patients (p <0.001). So, oxLDL increases with diabetes duration even with desirable LDL-cholesterol levels (18).

Garrido-Sanchez et al. showed a negative correlation between the anti-oxLDL antibodies and development of carbohydrate disorders which may reflect the blocking role of oxLDL immune complexes in kidney and cardiovascular disorders associated with diabetes (14).

It is noteworthy that all the patients with GDM enrolled our study were firstly diagnosed in 24-26 gestational weeks; so, our findings show that even short-term hyperglycemic periods which occur during GDM may be enough to increase the oxLDL levels. Indeed, in agreement with these results, it has been showed that increased duration and extent of glucose intolerance is associated with lower LDL oxidation rate (19). This is in contrast with what is concluded from Nakhjavani et al. study that oxLDL levels are related to diabetes duration (18).

On the other hand, increased oxLDL levels may accelerate the atherosclerosis and microvascular complications of diabetes such as nephropathy (20-22); therefore, despite most studies which evaluated the effects of GDM on the fetus and adverse pregnancy outcome, the importance of aptly diagnosis of GDM in maternal health may be concluded from the current study especially if similar results be achieved from future studies conducted in this regard.

In conclusion, our study found significant increase of oxLDL in pregnancies complicated by GDM. This may be of importance in stressing the role of short-term hyperglycemia in the formation of oxLDL during GDM.

## Note

***This article has been extracted from M.D.*** ***thesis*** ***(Sara Yassini)***

## References

[1] American College of O, Gynecologists Committee on Practice B-O (2001). ACOG Practice Bulletin. Clinical management guidelines for obstetrician-gynecologists. Number 30, September 2001 (replaces Technical Bulletin Number 200, December 1994). Gestational diabetes. Obstet Gynecol.

[2] American Diabetes Association (2004). Gestational diabetes mellitus. Diabetes Care.

[3] Ethier-Chiasson M, Forest JC, Giguère Y, Masse A, Marseille-Tremblay C (2008). Modulation of placental protein expression of OLR1: implication in pregnancy-related disorders or pathologies. Reproduction.

[4] Nakhjavani M, Asgharani F, Khalilzadeh O, Esteghamati A, Ghaneei A, Morteza A (2011). Oxidized low-density lipoprotein is negatively correlated with lecithin-cholesterol acyltransferase activity in type 2 diabetes mellitus. Am J Med Sci.

[5] Stephens JW, Gable DR, Hurel SJ, Miller GJ, Cooper JA, Humphries SE (2006). Increased plasma markers of oxidative stress are associated with coronary heart disease in males with diabetes mellitus and with 10-year risk in a prospective sample of males. Clin Chem.

[6] Tsuzura S, Ikeda Y, Suehiro T, Ota K, Osaki F, Arii K (2004). Correlation of plasma oxidized low-density lipoprotein levels to vascular complications and human serum paraoxonase in patients with type 2 diabetes. Metabolism.

[7] Woodman RJ, Watts GF, Playford DA, Best JD, Chan DC (2005). Oxidized LDL and small LDL particle size are independently predictive of a selective defect in microcirculatory endothelial function in type 2 diabetes. Diabetes Obes Metab.

[8] Ben-Haroush A, Yogev Y, Hod M (2004). Epidemiology of gestational diabetes mellitus and its association with Type 2 diabetes. Diabetic medicine: Diabet Med.

[9] Otero P, Herrera E, Bonet B (2002). Dual effect of glucose on LDL oxidation: dependence on vitamin E. Free Radic Biol Med.

[10] American Diabetes A (2013). Standards of medical care in diabetes--2013. Diabetes Care.

[11] Toescu V, Nuttall SL, Martin U, Nightingale P, Kendall MJ, Brydon P (2004). Changes in plasma lipids and markers of oxidative stress in normal pregnancy and pregnancies complicated by diabetes. Clin Sci (Lond).

[12] Kanne JP, Lalani TA, Fligner CL (2005). The placenta revisited: radiologic-pathologic correlation. Curr Probl Diagn Radiol.

[13] Ethier-Chiasson M, Forest JC, Giguere Y, Masse A, Marseille-Tremblay C, Levy E (2008). Modulation of placental protein expression of OLR1: implication in pregnancy-related disorders or pathologies. Reproduction.

[14] Sanchez-Vera I, Bonet B, Viana M, Quintanar A, Martin MD, Blanco P (2007). Changes in plasma lipids and increased low-density lipoprotein susceptibility to oxidation in pregnancies complicated by gestational diabetes: consequences of obesity. Metabolism.

[15] Levitan I, Volkov S, Subbaiah PV (2010). Oxidized LDL: diversity, patterns of recognition, and pathophysiology. Antioxid Redox Signal.

[16] Scazzocchio B, Vari R, D'Archivio M, Santangelo C, Filesi C, Giovannini C (2009). Oxidized LDL impair adipocyte response to insulin by activating serine/threonine kinases. J Lipid Res.

[17] Gokulakrishnan K, Deepa R, Velmurugan K, Ravikumar R, Karkuzhali K, Mohan V (2007). Oxidized low-density lipoprotein and intimal medial thickness in subjects with glucose intolerance: the Chennai Urban Rural Epidemiology Study-25. Metabolism.

[18] Nakhjavani M, Khalilzadeh O, Khajeali L, Esteghamati A, Morteza A, Jamali A (2010). Serum oxidized-LDL is associated with diabetes duration independent of maintaining optimized levels of LDL-cholesterol. Lipids.

[19] Schwenke DC, D'Agostino RB Jr, Goff DC Jr, Karter AJ, Rewers MJ, Wagenknecht LE (2003). Differences in LDL oxidizability by glycemic status: the insulin resistance atherosclerosis study. Diabetes Care.

[20] Brownlee M (2005). The pathobiology of diabetic complications: a unifying mechanism. Diabetes.

[21] Nakhjavani M, Esteghamati A, Khalilzadeh O, Asgarani F, Mansournia N, Abbasi M (2010). Association of macroalbuminuria with oxidized LDL and TGF-beta in type 2 diabetic patients: a case-control study. Int Urol Nephrol.

[22] Renie G, Maingrette F, Li L (2007). Diabetic vasculopathy and the lectin-like oxidized low-density lipoprotein receptor-1 (LOX-1). Curr Diabetes Rev.

